# Analysis of COVID-19 Case Demographics and Disease Outcomes in Gary, Indiana

**DOI:** 10.3390/ijerph20186729

**Published:** 2023-09-07

**Authors:** Maryam Sabir, Yazan Al-Tarshan, Cameron Snapp, Martin Brown, Roland Walker, Amy Han, Tatiana Kostrominova

**Affiliations:** 1 Northwest Campus, Indiana University School of Medicine, Gary, IN 46408, USA; msabir@iu.edu (M.S.); yaltarsh@iu.edu (Y.A.-T.); camsnapp@indiana.edu (C.S.); 2 Gary Health Department, Gary, IN 46402, USA; martin@garysan.com (M.B.); rowalker@gary.gov (R.W.); 3 Department of Psychiatry, Indiana University School of Medicine, Indianapolis, IN 46202, USA; amyhan@iu.edu; 4 Department of Anatomy, Cell Biology and Physiology, Indiana University School of Medicine, Indianapolis, IN 46202, USA

**Keywords:** health disparities, COVID-19, Black population, income inequality, medically underserved community

## Abstract

Background: The COVID-19 pandemic further exposed the prevalence of existing health disparities in Black communities in the U.S. The current study evaluates COVID-19 data collected in Gary, Indiana, from June 2020 to June 2021. We hypothesized that the number of COVID-19 cases, hospitalizations, and deaths were influenced by race and income. Methods: In collaboration with the Gary Health Department (GHD), we analyzed demographic data on COVID-19-positive cases. Results: Compared to Gary’s non-Black population, age- and population-adjusted rates of hospitalizations and deaths in the Black population were 3-fold (*p* < 0.0001) and 2-fold (*p* < 0.05) higher, respectively. This is despite a higher infection rate (*p* < 0.0001) in the non-Black population. The median household income of a zip code was negatively correlated with COVID-19 hospitalizations (R^2^ = 0.6345, *p* = 0.03), but did not correlate with infections and deaths. Conclusions: The current study demonstrates clear health disparities of income and race in the context of COVID-19-related infections and outcomes in the city of Gary. Indiana University School of Medicine Northwest and GHD officials can collaborate to utilize these data for the reallocation of resources and health education efforts in Gary’s highly populated, low-income, and predominantly Black neighborhoods. It should also prompt further investigation into national health resource allocation.

## 1. Introduction

The COVID-19 pandemic had a devastating effect on numerous countries across the globe. It put a severe strain on health facilities and economies [1] and highlighted racial and socioeconomic health disparities. Marked by uncertainty, attempts at addressing the outbreak initially led to international lockdowns and large-scale quarantines without clear end dates. Increased cases of acute trauma or post-traumatic stress disorder during the pandemic demonstrate the severe impact of the mortality that ensued, in combination with economic instability and inevitable long-term lifestyle changes [2]. There is a large body of compelling evidence supporting the social determinants of health data as a strong predictor of health outcomes [3,4], and COVID-19 is not an exception. The review of the existing literature clearly shows the presence of COVID-19-related racial disparities in the United States, specifically in Black and impoverished populations [5,6]. Black individuals account for 12.4% of the American population and 14.3% of deaths by COVID-19 in the U.S. [7,8]. In comparison, White Americans comprise 61.6% of the population and 59.7% of deaths by COVID-19. This disparity is multifactorial and includes determinants such as poverty, access to healthcare, nutrition, and pre-existing co-morbid conditions [6].

Social factors that exist in Black and other minority communities, such as historically based mistrust of medical professionals, are also crucial to consider [9]. In order to fully assess the impact of such factors, it is important to understand both their development over time and their roles in individuals’ present daily lives. Historic experimentation through unethical and non-consensual means on Black individuals in medical research in the U.S. include well-known examples, such as the infamous Tuskegee Syphilis Study [10]. The non-consensual use of tissue samples belonging to Henrietta Lacks and the subsequent lack of consent demonstrated by the publication of her genome and widespread distribution of her cell lines is another historic example [11]. For Indigenous communities, research studies are often based on settler colonial agendas that reinforce harmful stereotypes and serve as a source of medical mistrust in Native populations [12]. The implications of medical mistrust remain deeply rooted in the present day. For example, it has been shown that Black men with more frequent exposure to everyday racism and perceived racism in health care are more likely to delay preventative health screenings [13]. Vaccine hesitancy and lower COVID-19 vaccination rates for the Black population in the U.S. are also significant contributing factors to disparities in COVID-19 impact [14,15]. Access to vaccinations is only one aspect of this issue, as medical mistrust and misinformation regarding the vaccine may also contribute to a lower vaccine administration. With a clear role in present-day attitudes and health behaviors, it is crucial to understand historically based medical mistrust in Black and minority communities to effectively contextualize active disparities.

Previous studies support the idea of a Matthew effect by which pandemic instability widens inequality by disproportionately affecting groups that are already disadvantaged [16]. In addition, cultural norms in predominantly Black communities that promote collective engagement through larger and more frequent group gatherings are associated with increased rates of COVID-19 diagnosis [17]. Environmental and geographic differences, such as urban environments, are also important to consider. Studies utilizing Geographic Information System (GIS) have found factors such as population density and asthma, suggestive of urban areas, to be related to higher rates of death by COVID-19 [18]. Spatial factors in urban environments that influence mobility and air quality have also been implicated with aggregates of COVID-19 cases [19].

Given the multi-factorial nature of health disparities, it is imperative to assess them at all levels ranging from local communities to a national scale. Documenting and evaluating the racial and economic disparities related to COVID-19 in particular is crucial for improving healthcare policy, assistive programs, and resource allocation at the local, state, and federal levels of affected communities. The focus of this study is to analyze the effects of COVID-19 specifically in the city of Gary, Indiana. Gary serves as a microcosm for impoverished, majority non-White, and urban communities across America. There is a large Black population, a high number of residents living below the poverty line, and a high unemployment rate [20]. The city is one of many in the U.S. that were severely impacted by deindustrialization. The closure of U.S. steel’s largest manufacturing plant, Gary Works, in the 1960s left the city to economic collapse. Compounded with discriminatory housing policies from the remnants of segregation and Jim Crow laws, the population split as White Americans fled the city to find new jobs [21].

According to the 2020 Census, the total population in Gary is 69,093 people and 78% of the population is Black, compared to 13% nationally. The mean household income of Gary residents is USD 34,085, which is well below the national average of USD 69,717 [20]. Both the unemployment rate (44.4%) and the poverty rate (32%) in Gary are well above their respective national averages [20]. With the multitude of social determinants of health indices affecting Gary’s population, we sought to elucidate the association of these factors with the number of COVID-19 infections and the severity of the disease, including hospitalizations and deaths. We specifically assessed racial and economic disparities by utilizing an age-adjusted race correlation and a population-adjusted zip code correlation to compare race categories and mean household income to COVID-19 infections and severity outcomes. We hypothesized that the Black population in Gary and zip codes with a lower mean household income would have a stronger correlation with the number of COVID-19 infections, hospitalizations, and deaths.

## 2. Methods

### 2.1. Data Collection

COVID-19 data were collected from the Gary Health Department from 16 June 2020 to 7 June 2021. De-identified data from all 5149 positive cases included demographics (race, age, and zip code) and indicated disease progression (hospitalizations and deaths). Demographic data on Gary and its zip codes, including population, age distributions, racial distribution, and median household income, were collected from the U.S. Census Bureau [20].

COVID-19 data from GHD were re-binned to match Census age groups. African Americans in the city of Gary represent 79.8% of the population as compared to the 14.1% non-Hispanic White Americans. Since races other than Black are underrepresented in Gary, COVID-19 racial data were binned to include Black and non-Black. For the purpose of this study, non-Black includes Other, White, Multi-Race, Asian, and American Indian/Alaska Native.

COVID-19 cases with missing race data were omitted for the racial differences analysis. Omitted data represented 46.7% of cases. This issue is not unique to the current study since race is underreported in 35% of COVID-19 cases nationally [22].

### 2.2. Observed/Expected (O/E) Normalization

Observed/Expected (O/E) ratio normalization for the age distribution effects of COVID-19 prevalence and severity were calculated and adjusted for missing race data using the following formula:

O/E (observed/expected):

$$\frac{O}{E}=\frac{observed\;count}{expected\;count}$$

$$expected\;count=\frac{cases\;with\;known\;race}{total\;cases}\times \sum_{age\;group}\left({population\;size}_{age\;group}\times {rate}_{age\;group}\right)$$

O/E ratio effectively adjusts for population size, age distribution, and data with missing race. The O/E ratio is a commonly used method of data comparisons in the clinical setting [23].

### 2.3. Statistical Analysis

In the analysis of racial disparities, Pearson’s chi-squared test was used to determine statistical significance, defined as a two-sided *p*-value of 0.05 or less. In this analysis, the expected values were calculated using the same formula for the expected count used in Section 2.2:

$$expected\;values=\frac{cases\;with\;known\;race}{total\;cases}\times \sum_{age\;group}\left({population\;size}_{age\;group}\times {rate}_{age\;group}\right)$$

In the analysis of the zip code distribution data, linear regression was used to model differences in COVID-19 severity and zip code-related variables. Linear regression analysis was used to determine the significance of differences in the population. Since these zip codes are adjacent to each other, the Pearson correlation coefficient was used instead of R^2^ to describe covariance in order to avoid issues with the model assumptions.

Exponential linear regression analysis was used to determine the significance of income distribution by the zip codes on COVID-19 outcomes. O/E ratios were converted to a log scale, and then linear regression was performed on logged O/E ratios. Since these zip codes are adjacent to each other, the Pearson correlation coefficient was used instead of R^2^ to describe covariance in order to avoid issues with the model assumptions.

## 3. Results

### 3.1. Evaluation of the Number of Infections, Hospitalizations, and Deaths in the City of Gary

**Figure 1:**
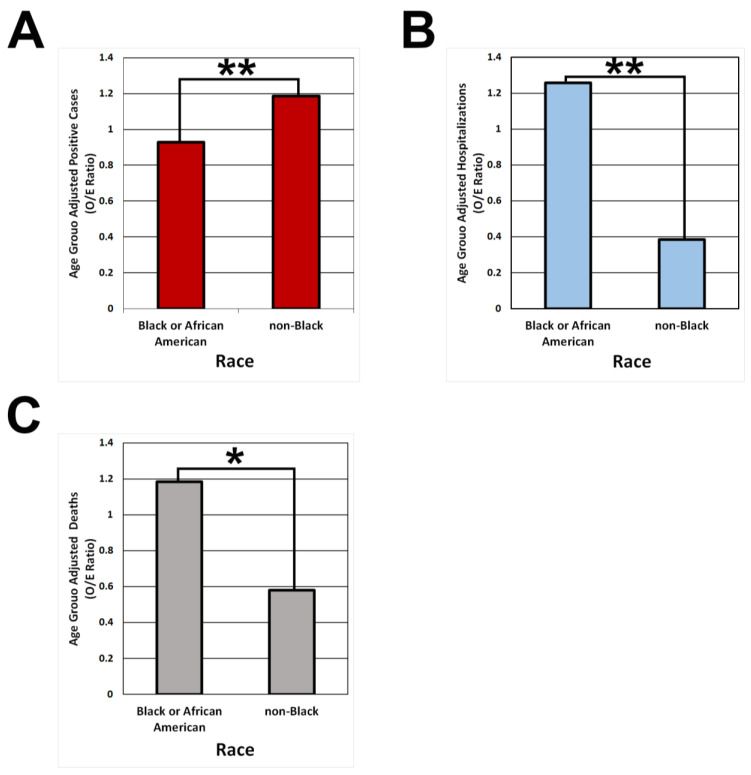
Age-group-adjusted O/E ratio of the number of infections, hospitalizations, and deaths in the city of Gary. Comparisons for the number of positive cases (**A**), hospitalizations (**B**), and deaths (**C**) for Black and non-Black populations are presented. Significance was determined by a Pearson’s chi-squared test. Asterisks represent statistically significant differences (* *p* < 0.05, ** *p* < 0.001).

The analysis of the number of positive cases showed that there was a higher number of infections in the non-Black population compared to the Black population (1.19 vs. 0.93, *p* < 0.0001; Figure 1A).

Despite lower infections, there was a statistically significantly higher number of COVID-19-related hospitalizations for the Black population compared to the non-Black population. After O/E normalization, there was a 3-fold higher number of COVID-19 hospitalizations in the Black versus the non-Black population (1.26 to 0.38, *p* < 0.0001; Figure 1B).

There also was a significantly higher number of age-adjusted deaths in the Black versus the non-Black population. After O/E normalization, there was a 2-fold higher number of COVID-19 hospitalizations in the Black versus the non-Black population (1.19 to 0.58, *p* < 0.02; Figure 1C).

### 3.2. Evaluation of the Effect of Zip Code Income on Infections, Hospitalizations, and Deaths in the City of Gary

**Figure 2:**
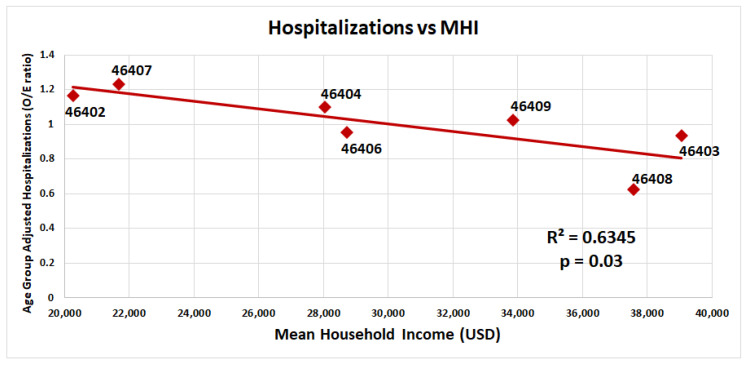
Age-group-adjusted COVID-19 hospitalizations versus median household income in Gary, Indiana, by a zip code. Age-group-adjusted hospitalizations (hospitalizations O/E ratio) of seven different zip codes (46402, 46403,46404, 46406, 46407, 46408, and 46409) in the city of Gary were plotted against median household income by the zip code (red square, USD/year). Trendline represents the linear regression (R^2^ = 0.63, *p* = 0.03). Significance was determined using a linear regression analysis.

Age-group-adjusted COVID-19 hospitalizations versus median household income of zip codes in Gary, Indiana, are presented in Figure 2. As the median household income of a zip code decreases, the number of hospitalizations increases.

**Figure 3:**
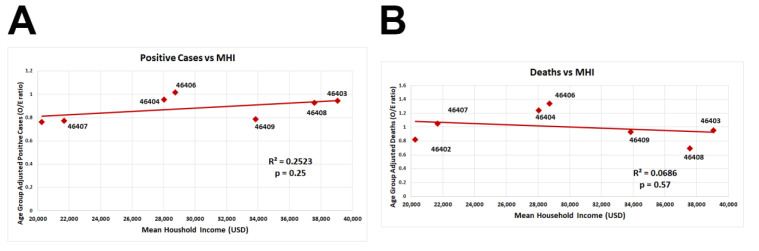
Age-group-adjusted COVID-19 number of positive cases and deaths versus median household income in Gary, Indiana, by zip code. Age-group-adjusted number of positive cases (**A**) and deaths (**B**) of seven different zip codes (46402, 46403,46404, 46406, 46407, 46408, and 46409) in the city of Gary were plotted against median household income by the zip code (red square, USD/year). Trendline represents the linear regression (A: R^2^ = 0.25, *p* = 0.25; B: R^2^ = 0.06, *p* = 0.57). Significance was determined using a linear regression analysis.

There were no correlations between zip code median household income and the number of positive cases or deaths (Figure 3A,B).

### 3.3. Infection Rates Correlate with the Zip Code Population Size

**Figure 4:**
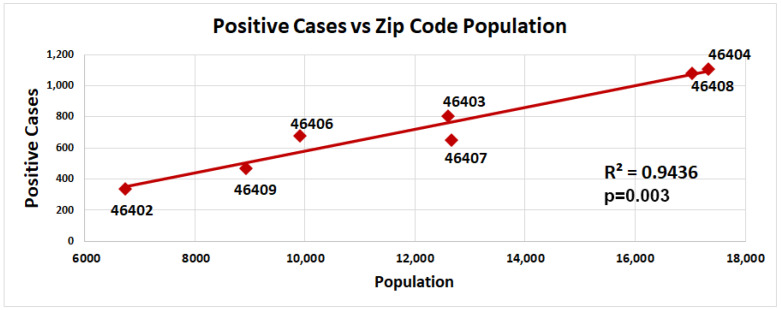
Number of COVID-19-positive cases correlates with the population size in Gary, Indiana, by zip code. Positive cases (number) of seven different zip codes (46402, 46403, 46404, 46406, 46407, 46408, and 46409) in the city of Gary were plotted against population size of the zip code (red circles, number of positive cases). Trendline represents the linear regression (ρ = 0.9436, *p* = 0.003). Significance was determined using a linear regression analysis.

As a zip code population size increased, the number of detected COVID-19 infections in that zip code also increased (Figure 4). This correlation was expected assuming that people in each zip code area had similar infection rates and COVID-19 testing availability. If any zip code area differed in either the availability of COVID-19 tests or infection rates, this would be reflected by a lack of correlation between the zip code population size and the number of COVID-19 infections.

## 4. Discussion

It has been documented in previous studies that COVID-19 disproportionality affects individuals of specific racial and economic demographics. The existing data demonstrate racial disparities in the impact of COVID-19, specifically in Black and impoverished populations in the United States [6,24]. This disparity is multifactorial and includes determinants such as poverty, access to healthcare, nutrition, and pre-existing co-morbid conditions.

The current study evaluated whether Black and non-Black populations in Gary, Indiana, were impacted differently by COVID-19. The effects of income differences on COVID-19 infections and outcomes between zip codes in Gary were also evaluated. Three demographic variables from the U.S. Census data were significantly correlated to COVID-19 prevalence and severity. First, population size impacted the number of positive tests. Second, racial disparities exist in the number of infections, hospitalizations, and deaths of the Black population versus the non-Black population in Gary. Third, COVID-19 hospitalizations are significantly and negatively associated with zip code income.

In this study, population size positively correlated with the number of COVID-19 cases, such that zip codes with greater populations had a higher number of infections. COVID-19 is transmitted from person to person by the direct inhalation of contaminated droplets or by close contact with an infected person [25]. In zip codes with larger populations, it is more difficult to practice social distancing and avoid close contact, increasing the likelihood of spreading COVID-19. Therefore, it is not surprising to find a higher number of positive cases in the more densely populated zip codes of Gary.

The current study demonstrated a racial disparity in the number of positive COVID-19 cases in Gary, Indiana. A statistically significant difference was identified in the number of COVID-19 infections between the Black and non-Black populations, with the non-Black population having a higher number of infections. This finding is unexpected and differs from what has been reported in some previous studies. For example, Black individuals in Indiana’s Marion County were four times more likely to test positive for COVID-19 than non-Black individuals. Counties with a higher Black population also had an increased number of positive COVID-19 cases [26]. One explanation for the dissimilarities between the current study and data reported by Hanson and colleagues [26] is that many testing centers in Gary became vaccination centers between the end of 2020 and the beginning of 2021. With fewer people seeking tests at the facilities, many were not tested until hospitalization when they became critically ill. This may explain the finding of the current study that the Black population has a lower infection rate, but a much higher hospitalization rate compared with the non-Black population.

A second plausible explanation as to why the non-Black population in Gary had a higher infection rate than the Black population is differences in resource availability. This difference in resources can be quantified by comparing the median household incomes for the two groups. As of 2020, the median household income for the Black population in the United States was USD 45,870, while the median household income for Hispanics and white non-Hispanics was USD 55,321 and USD 74,912, respectively [7]. Income inequality has been further exacerbated by the COVID-19 pandemic. Black adults were found to be over three times as likely as White adults to report food insecurity, being laid off, or being unemployed [16]. A lower median household income and the greater likelihood of losing employment are additional barriers to obtaining COVID-19 testing for the Black population compared to the non-Black population. When comparing the unemployment rates, the Gary zip code 46402 with one of the highest hospitalization values had the unemployment rate of 21.7%. The zip code 46408 with the lowest hospitalization values had the unemployment rate of 14.7%. The assumption that the non-Black population was obtaining tests at a much higher rate than the Black population in Gary would explain the higher infection rate that differs from the previous studies. This is also supported by a study conducted in Illinois, which reported the total number of confirmed cases among Black and White individuals as almost even, despite 220,968 White individuals being tested compared to only 78,650 Black individuals [6]. Based on these, it is possible that many positive cases in the Black population were undocumented.

The current study found racial disparities related to COVID-19 outcomes in Gary. A statistically significant difference was identified in the frequency of both hospitalizations and deaths between the Black and non-Black communities. There was a three-fold increase in the number of COVID-19 hospitalizations in the Black versus the non-Black population. There was also a two-fold increase in the number of COVID-19 deaths in the Black versus the non-Black population. These findings are consistent with evidence from previous studies. One study conducted at Montefiore Health System in the Bronx investigated hospitalizations and mortality rates of their Black and White patients prior to COVID-19 and during the pandemic. The study found that, during the COVID-19 period, the adjusted odds of death for Black patients were 1.6 times higher than that of White patients [27]. Another study researching racial disparities in COVID-19-related deaths across the entire United States found that only 20% of U.S. counties are disproportionately Black, despite accounting for 52% of COVID-19 diagnoses and 58% of COVID-19 deaths [28].

Pre-existing comorbidities also play an immense role in determining the severity of COVID-19 disease. Studies have found COVID-19 patients with cardiovascular disease, diabetes, congestive heart failure, hypertension, chronic kidney disease, and cancer to have a significantly higher risk of mortality compared to COVID-19 patients without these pre-existing comorbidities [29]. A higher prevalence of obesity, diabetes, hypertension, and chronic kidney disease has been shown in Black patients compared with White patients [30]. The Black population also experiences a drastically higher rate of food insecurity in the U.S. One study reported the rate of food insecurity in Indiana’s Black population after the COVID-19 pandemic (55%) to be 134% higher than that of the White population and 308% higher than the rate in Indiana prior to the pandemic (14%) [16]. It is clear that the disparity in food insecurity already experienced by the Black population became significantly worse during the pandemic [16]. The increase in food insecurity contributes to difficulties in lifestyle changes that may lead to the further exacerbation of pre-existing comorbidities. It can be reasonably suggested that the higher number of COVID-19 deaths in the Black population in Gary may be related to the greater prevalence of pre-existing comorbidities and diminished socioeconomic opportunities to adhere to a healthy lifestyle.

Some studies also point to vaccine hesitancy in the Black population contributing to a lower vaccination rate compared to the non-Black population in the U.S. The prevalence of COVID-19 vaccination hesitancy for adults in the U.S. was 26.3%, while the prevalence rate for the Black population was 41.6% [14]. There are multiple factors that could explain this significant difference in vaccine hesitancy [31,32]. Experience with racial discrimination and mistreatment in both healthcare and non-healthcare settings contributes to an increased vaccine hesitancy by patients developing medical mistrust [33]. Misinformation through mass media and social media is also a major contributor to vaccine hesitancy in minority communities [15]. Current data on vaccination support vaccination as a means to prevent contracting COVID-19 infection and significantly reduce the severity of the disease [34]. As the Black population receives the vaccine at a significantly lower rate, they are more likely to experience greater severity of symptoms and higher rates of mortality once they contract COVID-19. Vaccination hesitancy can be improved by providing culturally informed, consistent education and increasing access by distributing vaccines at local churches and community centers by trusted community members [15]. Furthermore, messaging to Black individuals from physicians of the same race has been shown to increase knowledge of COVID-19 symptoms and prevention methods [35].

The results from this study also found hospitalizations for COVID-19 to be considerably affected by income. A statistically significant correlation was found between the median household income of a zip code and the number of hospitalizations due to COVID-19 infection. Individuals in zip codes with a lower median household income in Gary are more likely to be hospitalized due to COVID-19 infection. Similar to the previous trends, there are multiple factors contributing to the finding of lower-income zip codes having more hospitalizations. A higher income offers a greater access to testing facilities and treatments, allowing patients to receive positive test results earlier and start treatment sooner [6]. Individuals with a lower income and less access to testing may not know of their infection until the disease significantly progresses and at which point hospitalization becomes inevitable. A higher income also offers a greater access to health insurance [36], preventative healthcare, and newer treatment options. In addition to beginning treatment relatively early, these patients may have a greater ability to take time off from work and impede the progression of the disease before hospitalization is required. Previous studies have found pre-pandemic food insecurity, housing insecurity, and unemployment status to be associated with most indicators of pandemic-driven economic instability [16]. These factors are likely to collectively contribute to a more severe disease progression and a greater likelihood of hospitalizations for individuals with a lower income.

Historic restrictions in access to education and digital technologies in minority communities might significantly contribute to the income inequalities and mistrust in healthcare professionals as well [37]. Both of these factors greatly influence the outcome of COVID-19 infection by impeding access to reliable information and technology to make informed healthcare decisions. A recent retrospective observational study utilizing the COVID-19 Research Database to analyze factors contributing to telehealth inequities showed that patients with high school education or less were less likely to use telehealth than those with a bachelor’s degree or higher [38]. This is important to consider in the context of the COVID-19 pandemic as telehealth visits became an increasingly popular way to access the healthcare system.

It is evident that numerous factors contribute to the existing racial health inequalities in Gary that are further highlighted by the COVID-19 pandemic. Overcoming these inequalities requires coordinated effort from multiple local, state, and national organizations. As it was described in previously published studies, clinicians, public health professionals and researchers can all play a role in the advancement of health equity for Black Americans and other racial and ethnic minority communities [24]. Further directions for the current study include investigating which socioeconomic disparities specifically have the greatest impact on COVID-related health outcomes. We discussed several factors that may have contributed to the disparities that were found in this study, including limited access to testing, lack of health insurance and decreased access to healthcare overall, pre-existing comorbid conditions, vaccine hesitancy, and food insecurity. While these factors collectively contribute to disparities in COVID-19 impact, elucidating the associations of each factor would help to form targeted strategies to mitigate their impact. In addition to providing valuable insights to guide higher-level policy and resource distribution, this information could help clinicians to risk-stratify patients in their clinical practice. Overall, the current findings and future directions have the potential to characterize which socioeconomic factors should be specifically targeted at multiple levels to augment measures aimed at addressing the existing healthcare disparities.

## 5. Conclusions

The current study showed that: (1) population size affected the number of positive cases; (2) racial disparities are present in the number of infections, hospitalizations, and deaths in the Black population versus the non-Black population in Gary; and (3) COVID-19 hospitalizations have a significant negative association with zip code income. The critical assessment and evaluation of these results can assist the Gary Health Department in creating a more targeted COVID-19 response to better address the needs of the Gary community. In addition to addressing COVID-19 impact, informed measures would also help to prevent the exacerbation of these disparities by future outbreaks.

It is important to note the stark disparities in COVID-19 impact between the neighboring zip codes found in this study. Negative differences in health outcomes for neighboring residents are concerning for factors that can be addressed by local interventions, and zip code data may serve as a strong starting point to develop these interventions [39]. As a microcosm for impoverished, majority non-White urban communities across the U.S., these findings should prompt further investigation by health departments into local trends to inform policy and resource allocation. Medical students and medical school faculty play a unique role in community health, and in collaboration with the local health departments, can promote health education in high-risk areas with strategically accessible vaccination sites, regular health screenings, and educational initiatives to promote overall healthy lifestyles. Campaigns to foster trust between medical professionals and community members would also aid in addressing hesitancy with vaccinations and other preventative or active treatment options. Connecting with grassroots organizations and community leaders is another way to build partnerships and promote sustainable change in minority communities. As research findings of studies on health disparities can be influential in the redistribution of preventative resources, novel treatments and educational initiatives to overcome health misinformation and mistrust and identifying and evaluating the causes of racial and economic disparities in healthcare are critical steps in addressing disparities in COVID-19 impact.

## Data Availability

The data presented in this study are available on request from the corresponding author. Data was obtained from the Gary Health Department and are available after approval of the Gary Health Department.

## References

[1] Jackson J.K., Weiss M.A., Schwarzenberg A.B., Nelson R.M., Sutter K.M., Sutherland M.D. (2021). Global Economic Effects of COVID-19. https://sgp.fas.org/crs/row/R46270.pdf.

[2] Chamaa F., Bahmad H.F., Darwish B., Kobeissi J.M., Hoballah M., Nassif S.B., Ghandour Y., Saliba J.P., Lawand N., Abou-Kheir W. (2021). PTSD in the COVID-19 Era. Curr. Neuropharmacol..

[3] Braveman P., Gottlieb L. (2014). The social determinants of health: It’s time to consider the causes of the causes. Public. Health Rep..

[4] Howell C.R., Harada C.N., Fontaine K.R., Mugavero M.J., Cherrington A.L. (2023). Perspective: Acknowledging a Hierarchy of Social Needs in Diabetes Clinical Care and Prevention. Diabetes Metab. Syndr. Obes..

[5] Reyes C., Husain N., Christy Gutowski S.S.C., Pratt G. (2020). Chicago’s coronavirus disparity: Black Chicagoans are dying at nearly six times the rate of white residents, data show. Chicago Tribune.

[6] Vasquez Reyes M. (2020). The Disproportional Impact of COVID-19 on African Americans. Health Hum. Rights.

[7] US Census Bureau (2020). 2020 Census Illuminates Racial and Ethnic Composition of the Country. https://www.census.gov/library/stories/2021/08/improved-race-ethnicity-measures-reveal-united-states-population-much-more-multiracial.html.

[8] CDC.gov (2023). Provisional COVID-19 Deaths: Distribution of Deaths by Race and Hispanic Origin. https://data.cdc.gov/NCHS/Provisional-COVID-19-Deaths-Distribution-of-Deaths/pj7m-y5uh.

[9] Rusoja E.A., Thomas B.A. (2021). The COVID-19 pandemic, Black mistrust, and a path forward. eClinicalMedicine.

[10] Kennedy B.R., Mathis C.C., Woods A.K. (2007). African Americans and their distrust of the health care system: Healthcare for diverse populations. J. Cult. Divers..

[11] Beskow L.M. (2016). Lessons from HeLa Cells: The Ethics and Policy of Biospecimens. Annu. Rev. Genom. Hum. Genet..

[12] Brockie T.N., Hill K., Davidson P.M., Decker E., Krienke L.K., Nelson K.E., Nicholson N., Werk A.M., Wilson D., Around Him D. (2022). Strategies for culturally safe research with Native American communities: An integrative review. Contemp. Nurse.

[13] Powell W., Richmond J., Mohottige D., Yen I., Joslyn A., Corbie-Smith G. (2019). Medical Mistrust, Racism, and Delays in Preventive Health Screening Among African-American Men. Behav. Med..

[14] Khubchandani J., Macias Y. (2021). COVID-19 vaccination hesitancy in Hispanics and African-Americans: A review and recommendations for practice. Brain Behav. Immun. Health.

[15] Martinez Leal I., Njoh J., Chen T.A., Foreman-Hays F., Reed B.C., Haley S.A., Chavez K., Reitzel L.R., Obasi E.M. (2023). Exploring COVID-19 Vaccine Attitudes among Racially and Ethnically Minoritized Communities: Community Partners’ and Residents’ Perspectives. Int. J. Environ. Res. Public Health.

[16] Perry B.L., Aronson B., Pescosolido B.A. (2021). Pandemic precarity: COVID-19 is exposing and exacerbating inequalities in the American heartland. Proc. Natl. Acad. Sci. USA.

[17] Ransome Y., Ojikutu B.O., Buchanan M., Johnston D., Kawachi I. (2021). Neighborhood Social Cohesion and Inequalities in COVID-19 Diagnosis Rates by Area-Level Black/African American Racial Composition. J. Urban. Health.

[18] Ramirez I.J., Lee J. (2020). COVID-19 Emergence and Social and Health Determinants in Colorado: A Rapid Spatial Analysis. Int. J. Environ. Res. Public. Health.

[19] Wang R., Liu L., Wu H., Peng Z. (2022). Correlation Analysis between Urban Elements and COVID-19 Transmission Using Social Media Data. Int. J. Environ. Res. Public. Health.

[20] US Census Bureau QuickFacts Gary City, Indiana. https://www.census.gov/quickfacts/garycityindiana.

[21] Davich J. (2015). Lost Gary, Indiana.

[22] The COVID Racial Data Tracker COVID-19 Is Affecting Black, Indigenous, Latinx, and Other People of Color the Most. https://covidtracking.com/race.

[23] Hamo C.E., Fonarow G.C., Greene S.J., Vaduganathan M., Yancy C.W., Heidenreich P., Lu D., Matsouaka R.A., DeVore A.D., Butler J. (2021). Temporal trends in risk profiles among patients hospitalized for heart failure. Am. Heart J..

[24] Johnson-Agbakwu C.E., Ali N.S., Oxford C.M., Wingo S., Manin E., Coonrod D.V. (2022). Racism, COVID-19, and Health Inequity in the USA: A Call to Action. J. Racial Ethn. Health Disparities.

[25] Esakandari H., Nabi-Afjadi M., Fakkari-Afjadi J., Farahmandian N., Miresmaeili S.M., Bahreini E. (2020). A comprehensive review of COVID-19 characteristics. Biol. Proced. Online.

[26] Hanson A.E., Hains D.S., Schwaderer A.L., Starr M.C. (2020). Variation in COVID-19 Diagnosis by Zip Code and Race and Ethnicity in Indiana. Front. Public. Health.

[27] Golestaneh L., Neugarten J., Fisher M., Billett H.H., Gil M.R., Johns T., Yunes M., Mokrzycki M.H., Coco M., Norris K.C. (2020). The association of race and COVID-19 mortality. eClinicalMedicine.

[28] Millett G.A., Jones A.T., Benkeser D., Baral S., Mercer L., Beyrer C., Honermann B., Lankiewicz E., Mena L., Crowley J.S. (2020). Assessing differential impacts of COVID-19 on black communities. Ann. Epidemiol..

[29] Ssentongo P., Ssentongo A.E., Heilbrunn E.S., Ba D.M., Chinchilli V.M. (2020). Association of cardiovascular disease and 10 other pre-existing comorbidities with COVID-19 mortality: A systematic review and meta-analysis. PLoS ONE.

[30] Price-Haywood E.G., Burton J., Fort D., Seoane L. (2020). Hospitalization and Mortality among Black Patients and White Patients with COVID-19. N. Engl. J. Med..

[31] Hu S., Xiong C., Li Q., Wang Z., Jiang Y. (2022). COVID-19 vaccine hesitancy cannot fully explain disparities in vaccination coverage across the contiguous United States. Vaccine.

[32] McCabe S.D., Hammershaimb E.A., Cheng D., Shi A., Shyr D., Shen S., Cole L.D., Cataldi J.R., Allen W., Probasco R. (2023). Unraveling attributes of COVID-19 vaccine acceptance and uptake in the U.S.: A large nationwide study. Sci. Rep..

[33] Dong L., Bogart L.M., Gandhi P., Aboagye J.B., Ryan S., Serwanga R., Ojikutu B.O. (2022). A qualitative study of COVID-19 vaccine intentions and mistrust in Black Americans: Recommendations for vaccine dissemination and uptake. PLoS ONE.

[34] Lin D.Y., Gu Y., Wheeler B., Young H., Holloway S., Sunny S.K., Moore Z., Zeng D. (2022). Effectiveness of COVID-19 Vaccines over a 9-Month Period in North Carolina. N. Engl. J. Med..

[35] Alsan M., Stanford F.C., Banerjee A., Breza E., Chandrasekhar A.G., Eichmeyer S., Goldsmith-Pinkham P., Ogbu-Nwobodo L., Olken B.A., Torres C. (2021). Comparison of Knowledge and Information-Seeking Behavior After General COVID-19 Public Health Messages and Messages Tailored for Black and Latinx Communities: A Randomized Controlled Trial. Ann. Intern. Med..

[36] Doty M.M., Holmgren A.L. (2004). Unequal Access: Insurance Instability among Low-Income Workers and Minorities.

[37] Jaiswal J., Halkitis P.N. (2019). Towards a More Inclusive and Dynamic Understanding of Medical Mistrust Informed by Science. Behav. Med..

[38] Williams C., Shang D. (2023). Telehealth Usage Among Low-Income Racial and Ethnic Minority Populations During the COVID-19 Pandemic: Retrospective Observational Study. J. Med. Internet Res..

[39] Arrieta M., White H.L., Crook E.D. (2008). Using zip code-level mortality data as a local health status indicator in Mobile, Alabama. Am. J. Med. Sci..

